# Workplace risk assessment criteria for pregnant workers exposed to physical exertion and biological and chemical hazards

**DOI:** 10.2478/aiht-2025-76-3996

**Published:** 2025-06-30

**Authors:** Tea Samardžić, Roko Žaja, Željka Babić, Jelena Macan

**Affiliations:** PROTEGO Healthcare Institution, Koprivnica, Croatia; University of Zagreb School of Medicine, Andrija Štampar School of Public Health, Zagreb, Croatia; Institute for Medical Research and Occupational Health, Division for Occupational and Environmental Health, Zagreb, Croatia

**Keywords:** biological agents, CLP Regulation, expert opinion, foetal health, Key Indicator Method, maternal health, physical workload, reprotoxic substances, biološki agensi, fizičko preopterećenje, metoda ključnih pokazatelja, mišljenje stručnjaka, reprotoksične tvari, Uredba CLP, zdravlje fetusa, zdravlje majke

## Abstract

Workplace risk assessment criteria for pregnant workers in the EU remain inconsistent and poorly harmonised, with notable gaps in practical guidelines for occupational health physicians (OHPs). This regulatory ambiguity could lead to either insufficient protection or unnecessary exclusion of pregnant workers from the workplace, with significant implications for maternal and foetal health, as well as healthcare and social security systems. The aim of this study was to propose common, harmonised criteria for workplace risk assessment in healthy pregnant workers exposed to physical exertion and biological and chemical hazards. The criteria were developed through structured expert consultation involving occupational and sports medicine specialists from the Croatian Society of Occupational Health. To that end, we compiled and presented relevant legal and scientific literature, which served as the basis for discussion. Expert opinion was obtained via an anonymous online questionnaire administered during a structured expert workshop. The proposed criteria are based on the CLP Regulation (EC No. 1272/2008) classification of reprotoxic substances, identification of key biological hazards (e.g., cytomegalovirus, parvovirus B19, rubella virus, varicella-zoster virus, and *Toxoplasma gondii*), and assessment of physical workload using the Key Indicator Methods (KIM) developed by the German Federal Institute for Occupational Safety and Health (BAuA). By integrating legal context, medical evidence, and expert judgment, the proposed criteria aim to support consistent, timely, and evidence-based risk assessment and to facilitate national and EU guideline development for the protection of pregnant workers.

## Abstract (hr)

Kriteriji procjene rizika na radnome mjestu za trudnice unutar Europske unije nedovoljno su jasno definirani i slabo usklađeni, uz izražene nedostatke u praktičnim smjernicama za liječnike medicine rada. Nejasna pravna regulativa može rezultirati nedovoljnom zaštitom trudnica ili njihovim neopravdanim isključivanjem s radnog mjesta, što ima značajne posljedice za zdravlje trudnice i ploda, kao i na zdravstveni i socijalni sustav. Cilj ovoga rada bio je doprinijeti usklađivanju praksi stručnjaka predlaganjem kriterija za procjenu rizika na radnome mjestu za zdrave trudnice izložene fizičkom naporu te biološkim i kemijskim štetnostima. Prijedlog je razvijen tijekom rasprave sa specijalistima medicine rada i sporta – članovima Hrvatskoga društva za medicinu rada. Prije rasprave autori su prikupili te zatim predstavili relevantne zakonske i znanstvene izvore, koji su poslužili kao temelj za izradu prijedloga. Mišljenja stručnjaka prikupljena su putem anonimne ankete, provedene tijekom strukturirane rasprave. Predloženi kriteriji temelje se na klasifikaciji reprotoksičnih tvari prema Uredbi CLP (EZ br. 1272/2008), identifikaciji ključnih bioloških čimbenika rizika (npr. citomegalovirus, parvovirus B19, virus rubeole, virus *varicella-zoster*, *Toxoplasma gondii*) te procjeni fizičkog opterećenja metodom ključnih pokazatelja (engl. *key indicators method*, krat. KIM). Integriranjem pravnog okvira, medicinskih dokaza i stručne prosudbe, cilj je predloženih kriterija omogućiti pravovremenu, ujednačenu i na dokazima utemeljenu procjenu rizika te olakšati izradu smjernica za zaštitu trudnica na nacionalnoj i europskoj razini.

Maternity protection is a constitutional category in Croatia and includes protection of pregnant workers as a sensitive population group by Articles 24 and 25 of the Maternity and Parental Benefits Act and its bylaw (1), which adopts the 1992 Pregnant Workers EU Directive (2). According to this act, a pregnant worker whose job involves potential harm to her and/or the child's health is entitled to protection from these harms. The Maternity and Parental Benefits Act also stipulates that work in night shifts should be assessed for pregnant worker. If the employer has not ensured safe working conditions by either implementing appropriate occupational safety measures or by reassigning the worker to a new safe environment, pregnant workers are entitled to a pregnancy leave with salary compensation in the amount of the average salary paid in the last three months at the expense of the employer.

Jobs that may be harmful to the health of a pregnant worker or their child are assessed by an authorised occupational health physician (OHP) against the legal framework for the protection of pregnant workers, which also includes the bylaw on the safety and health protection of pregnant workers (3) arising from the Occupational Safety and Health Act (4). The latter determines risky jobs for a pregnant worker and jobs that may not be performed by a pregnant worker under any circumstances. Certain jobs are additionally defined as contraindicated for pregnant workers by the Ordinance on Jobs with Special Working Conditions (5).

Since workplace risks for a pregnant worker are assessed by OHPs, it is necessary to consolidate risk assessment criteria based on medical knowledge, scientific evidence, and the applicable legal frameworks. Our previous findings (6) show that such assessments for pregnant healthcare workers in Croatia lack clear guidance, forcing pregnant workers to misuse the healthcare system.

The aim of this paper is to address this issue and propose assessment criteria regarding workplace exposure of pregnant workers to physical exertion, biological hazards, and chemical hazards that all OHPs will support.

## DATA COLLECTION

To develop criteria for assessing workplace risks for pregnant workers by OHPs, we collected data from relevant legal and medical literature described below for biological and chemical hazards and physical exertion, as these hazards are considered the most relevant and demanding for pregnant workers. The proposed criteria are limited to healthy pregnant workers and pregnancies.

## EXPERT OPINION

The next step was to get feedback on the proposed criteria from OHPs. We took the opportunity to do that at the March 2025 workshop for OHP and sports medicine specialists, organised by the Croatian Society of Occupational Health (CSOH). At the beginning of the workshop, participants were informed about the proposed assessment criteria based on collected data. We took the opportunity to discuss the relationship between workplace risks and undesirable outcomes for pregnancy, acceptable risk levels at the workplace, and to propose absolute and relative contraindications to work for a pregnant worker. After the discussion, the participants were invited to answer our anonymous online questionnaire using mobile phones. Six questions were about the acceptability of workplace risks for pregnancy, and one was about the role of OHPs in assessing workplace risks for pregnancy. All answers were multiple-choice (I agree, I disagree, and I'm not sure), and each participant could answer each question once. The questions were answered by 20–37 participants, depending on the question. This makes 11.7–21.6 % of all CSOH members (171 at the time).

With the received feedback we were able to complete drafting the proposal for workplace risk assessment criteria for pregnant workers.

## PROPOSED WORKPLACE RISK ASSESSMENT CRITERIA FOR PREGNANT WORKERS

### Chemical hazards

#### Legal framework

Regarding reproductive toxicants, the European Commission (EC) Regulation on Classification, Labelling and Packaging (CLP) (No. 1272/2008) (7) aims to ensure safe use and handling and ultimately a high level of health protection by specifying labelling requirements that will provide information about possible harmful effects of hazardous substances and mixtures in raw materials or products. The main elements of categorisation and labelling of reproductive toxicants are given in Table 1. Simply put, lower category indicates greater hazard (7, 8). Category 1A denotes chemicals known to have adverse effects on foetal development in humans, largely based on evidence from humans. Category 1B denotes chemicals presumed to have adverse effects on foetal development in humans, largely based on reliable evidence from experimental animals, and category 2 denotes chemicals suspected to have adverse effects on development in humans based on evidence from humans or experimental animals which is not sufficient to justify 1A or 1B categorisation. Such evidence usually comes from studies of lower quality, whose design is wanting or the results are not clear enough to make strong conclusions. It also comes from studies with strong evidence of adverse effects in experimental animals but doubtful relevance for humans based on the mechanism of reproductive toxicity.

**Table 1:** Labelling elements for products/materials which can damage the unborn child (7)

**Pictogram**	**Hazard category**	**Hazard statement codes**	**Hazard statement text**
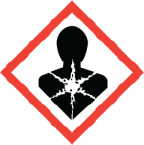	Reproductive toxicity 1A,1B	H360**D**	**May damage the unborn child.**
		H360F**D**	May damage fertility. **May damage the unborn child.**
		H360**D**f	**May damage the unborn child.** Suspected of damaging fertility.
		H360F**d**	May damage fertility. **Suspected of damaging the unborn child.**
	Reproductive toxicity 2	H361**d**	**Suspected of damaging the unborn child.**
		H361f**d**	Suspected of damaging fertility. **Suspected of damaging the unborn child.**

**Important note: in cases of combined effects**, e.g. may damage fertility (category 1) and suspected of damaging the unborn child (category 2), the more severe effect is used for the overall category of a product/chemical (in this case reproductive toxicity 1, accompanied by the statement H360Fd). **It is important to read the full text of the hazard statement, not just the classification**, to be informed if an effect on unborn child is expected or just suspected

**Table 2:** Seronegativity among women of reproductive age and the risk of foetal transmission if primary infection occurs (34,35,36,37,38)

**Pathogen**	**Estimated seronegativity among women of reproductive age**	**Risk of foetal transmission if primary infection occurs**
Cytomegalovirus	40–70 %	~30–40 % (severe sequelae in ~10 % cases)
Parvovirus B19	35–50 %	~30 % (associated with foetal hydrops)
Rubella virus	<5 % in vaccinated populations; <10 % without vaccination	up to 90 % in early pregnancy
Varicella-zoster virus	~5–10 % in developed countries	~2 % if infection occurs before 20 weeks
*Toxoplasma gondii*	40–60 %	increases with gestational age (early infections cause more severe foetal damage)

Categorisation also depends on whether it is a raw material (one chemical) or a finished product (a chemical mixture). For a single chemical, it is on the manufacturer or importer to propose classification and labelling (C&L) based on all available human and experimental evidence. After a thorough assessment by the Committee for Risk Assessment (RAC) at the European Chemicals Agency (ECHA) and a period of public consultation, the proposed C&L is modified as needed. Once it receives the final RAC opinion, the European Commission prepares a “harmonised” C&L for the substance of concern (9). Such harmonised C&L is the most reliable information on hazardous effects of any single chemical. As for chemical mixtures, they can be categorised automatically as 1A or 1B if the mixture contains ≥0.3 % of ingredients whose reproductive toxicity is category 1A or 1B, respectively, or as category 2 if it contains ≥3 % of category 2 ingredients. As new knowledge becomes available thanks to improvements in the methods for testing the reproductive toxicity of chemicals, their classification may be updated through an Adaptation to Technical Progress (ATP), which the European Commission issues every year in the form of Annex VI to the CLP Regulation (10). Lastly, in addition to European legislation, classification as toxic to reproduction is also available in relevant Croatian bylaws (11, 12).

Given the vast number of chemicals on the European market (around 350,000 single chemicals), and even more chemical products (13), and the fact that harmonising and updating the classification of even a single chemical takes years, whereas OHPs have the eightday deadline to give their risk assessment regarding a pregnant worker, here we relied only on the European and national legislation regarding single chemicals (7, 11, 12) and C&L information given in Safety Data Sheets for chemical products (14).

#### Proposed risk assessment criteria

Regarding chemical hazards, we propose that:

- the risk for the unborn child is acceptable if a chemical product or raw material is not labelled with any hazard statement communicating effects on unborn child (H360D, H360FD, H360Df, H360Fd, H361d, or H361fd);
- the risk for the unborn child is unacceptable (an absolute contraindication) if the mother works with chemical products or raw materials classified as Category 1A or 1B reprotoxic and labelled with a hazard statement communicating damage to unborn child (H360D, H360FD, or H360Df);
- the risk for the unborn child is acceptable if the possibility of damage for the unborn child is only suspected (H360Fd, H361d, or H361fd), but appropriate risk mitigation measures are implemented by the employer to bring exposure to the lowest possible level so that a moderate risk becomes negligible. However, if the protection measures (such as fume hood, ventilation systems, or personal protective equipment) are inadequate and inhalation exposure is relevant, then the risk is deemed unacceptable and work in such conditions contraindicated.

This proposal was supported by 18 workshop participants, rejected by six, while six were unsure. Those who were against said that they would not trust an employer to implement protective measures needed to bring the risk to a negligible level and would find the risk acceptable only if a chemical product or raw material is not labelled with any hazard statement communicating effects on unborn child.

According to the proposed algorithm for collecting data needed to assess the risk, the employer should submit to an OHP:

- the statement from the workplace risk assessment document;
- list of all products or raw materials with which a pregnant worker works, including chemical names and EC C&L, according to the CLP Regulation (EC) No. 1272/2008 and/or Safety Data Sheets;
- annual consumption of chemicals;
- estimated daily/weekly/monthly inhalational exposure;
- description of the work process;
- description of personal protective equipment and implemented technical and organisational risk mitigation measures;
- results of air concentration measurements in the working environment if available.

The OHP should also consider taking a tour of the workplace. If C&L data are not submitted, the OHP can check the Safety Data Sheets (SDS) of chemical products in the national SDS Registry (14) using the commercial names of the products provided by the employer. The C&L information with hazard statements is available in Section 2 Hazards Identification. Alternatively, the classification of a single chemical can be checked in ECHA's C&L inventory (13) or in national bylaws (11, 12) using the chemical name or unique numerical identifier (e.g. CAS number) provided by the employer. If a chemical is found to be a reproductive toxicant, the employer should be pressed to provide the SDS with a complete hazard statement to check if classification refers to effects on unborn child. In conclusion, we recommend combining all available sources of information, and, in case of doubt or contradictory data, consulting the national poison control centre.

We have limited our considerations of hazardous chemicals to reprotoxic chemicals with harmful effects to unborn child, which excludes harmful effects on fertility and lactation. The main issue with our proposed algorithm is the relevance of the reproductive toxicity category 2. Could a risk be considered negligible if an adverse effect is only suspected but not confirmed in humans, and appropriate risk mitigations measures are implemented to minimise exposure? The recommended technical and organisational measures, especially during tasks associated with high exposure (e.g., maintenance, equipment inspection, repairs, and so on), as well as health surveillance and management of medical records are stipulated by EU directives and national ordinances (11, 12, 15, 16). These measures, although reasonable, raise further questions in practice. Measurements of air concentration alone should never be the sole basis for assessment if inhalational exposure is acceptable. A recent study (17) has shown that only half of known or presumed reprotoxic chemicals (categories 1A and 1B) have an exposure limit. As the methodology for toxicity assessment improves, classification of certain chemicals may change to a more hazardous category (18). Uncertainties arise when animal data translated to human population are factored into occupational limits, as there is always a possibility of unexpected high susceptibility of the human foetus. In general, efforts are being made to gradually replace substances of very high concern (SVHC) with less dangerous substances or procedures where technically and economically feasible. At the level of the European Union, SVHC substances include reproductively toxic substances of category 1A or 1B and are therefore on the list of chemicals proposed for replacement (19). Generally, data on the reproductive toxicity of chemicals are considered insufficient on the EU level, which is why Claessens et al. (20) urge for better risk assessment and implementation of the precautionary principle in cases of exposure to category 2 chemicals.

### Biological hazards

#### Legal framework

In the EU and Croatia, reproductive biological hazards are categorised into four risk groups according to their pathogenicity, transmissibility, and the availability of effective prophylaxis or treatment. Group 1 biological agents are deemed unlikely to cause human disease. Group 2 agents can cause human disease but are unlikely to spread to the community and have generally effective prophylaxis or treatment. Group 3 agents cause serious disease, present a significant occupational hazard, and may spread to the community but usually have available effective preventive or therapeutic measures. Group 4 agents cause life-threatening diseases, pose a high risk of widespread transmission, and have no effective prophylaxis or treatment (21, 22). Exposure to group 4 agents poses an unacceptable risk for pregnant workers, and here selected groups 2 and 3 agents are relevant for risk assessment in pregnant workers.

Cytomegalovirus (CMV) is a group 2 herpesvirus transmitted via bodily fluids such as saliva, urine, genital secretions, and breast milk. Infection is common during childhood or adolescence, especially among individuals in close contact with young children. After primary infection, CMV establishes lifelong latency with potential reactivation. Consequently, a high proportion of women of reproductive age exhibit CMV-specific IgG antibodies, with estimated seroprevalence ranging from 63 % to 76 % in the WHO European region (23).

Parvovirus B19 is a group 2 agent responsible for *erythema infectiosum*, primarily transmitted via respiratory droplets and blood. Natural infection typically occurs during childhood, resulting in approximately 70 % seroprevalence among women of reproductive age demonstrating immunity (24). Parvovirus B19 carries a moderate to high risk, especially during seasonal outbreaks in school settings.

*Rubivirus rubella* is a group 2 agent, whose nearly general seropositivity is largely owed to extensive vaccination with the combined measles-mumps-rubella (MMR) vaccine. Before this extensive immunisation, rubella infection was nearly universal in children (25, 26).

Varicella-zoster virus (VZV) is a group 2 agent causing primary varicella (chickenpox) during childhood, and reactivated herpes zoster (shingles) later in adulthood, typically in temperate regions. Its seropositivity exceeds 90 % among adults. After primary infection, VZV persists in a latent state within sensory ganglia, with specific IgG antibodies present for life (27, 28).

VZV and rubella virus pose significant threats when immunity is lacking, with rubella being particularly dangerous because of its potential to cause severe congenital malformations. Occupational settings such as childcare facilities, educational institutions, and healthcare environments are known breeding grounds for these infections (29, 30).

*Toxoplasma gondii* is a group 2 intracellular protozoan parasite acquired mainly through ingestion of undercooked meat, exposure to contaminated soil, or contact with cat faeces. Infection is often asymptomatic, and seroprevalence varies widely, depending on dietary habits, hygienic standards, and geographic factors. It is typically encountered in agricultural work, handling of raw meat, or contact with contaminated soil. The presence of IgG antibodies indicates past infection and protects against congenital transmission during future pregnancies (31, 32).

The impact of an infectious disease on pregnancy differs substantially and depends on whether the maternal infection is primary or recurrent. Primary infections with pathogens such as CMV, rubella virus, VZV, parvovirus B19, and *T. gondii* are associated with a high risk of foetal transmission and serious outcomes, including miscarriage, congenital anomalies, intrauterine growth restriction, and foetal death. The absence of pre-existing maternal immunity allows pathogens to cross the placenta more readily and affect foetal development during critical periods. In contrast, subsequent infections, whether through viral reactivation or reinfection with a new strain, generally carry a significantly lower risk for the foetus. Pre-existing immunity, manifested through memory B and T cell responses, typically limits systemic dissemination of the pathogen and reduces the viral or microbial load, thereby decreasing the likelihood and severity of transplacental transmission (33,34,35,36,37,38).

*Listeria monocytogenes* is a group 2 agent primarily associated with food production. Even though the risk of infection is low to moderate, poor hygienic conditions can increase it significantly. Pregnant women are 16–18 times more likely to get infected from contaminated food than non-pregnant women. Infection in the first trimester carries a 65 % risk of miscarriage, while in the second or third trimester the risk drops to about 26 % (39).

*Coxiella burnetii* is a group 3 causative agent of Q fever, bearing a moderate to high risk in agricultural settings, particularly in farms involving sheep, goats, and cattle, where exposure to birth products (e.g., placenta, amnion, chorion, amniotic and allantoic fluid, and umbilical cord) can be significant. Q fever can cause inflammation of the placenta and direct infection of foetal organs, leading to low birth weight, premature birth, or miscarriage. It may also increase the mother's risk of developing chronic Q fever (40).

According to the recent Spanish guidance for assessing occupational risk during pregnancy (41), the assessment of serological status and workplace exposure to biological agents must be performed concurrently and systematically to ensure effective protection of pregnant workers and their unborn children. The guidance establishes that both immune status and the presence or likelihood of occupational exposure are critical in determining the acceptability of biological risks during pregnancy. When serological testing demonstrates immunity against a specific pathogen and there is no significant workplace exposure to that agent, the occupational risk is considered acceptable. Conversely, if serological testing reveals susceptibility (seronegativity) or if serological data are unavailable and occupational exposure is confirmed or probable, the risk must be classified as unacceptable, necessitating reassignment or enhanced protective measures. In cases where only one piece of information is available – for example, known immune status but uncertain exposure or confirmed exposure but unknown immune status – the guidance advocates for the precautionary principle. In such instances, the pregnant worker must be presumed at risk, and occupational exposure should be managed as if susceptibility were confirmed until definitive information becomes available. Where neither serological data nor exposure assessment is available, the guidance mandates that the risk must automatically be classified as unacceptable. The worker must be given protection equivalent to that required for a susceptible individual with confirmed exposure.

### Proposed risk assessment criteria

For healthcare workers caring for young children, childcare workers, and animal handlers we propose the following assessment regarding exposure to biological hazards:

- the risk is unacceptable for all pregnant workers with any exposure to group 4 biological agents;
- the risk is acceptable in case of exposure to group 2 and 3 biological agents with applied regular protective framework (hygiene procedures, personal protective equipment).

Our OHP panel was invited to evaluate our risk assessment proposals against real-world workplace scenarios, and a significant minority either disagreed or remained uncertain in response to the proposed risk acceptability for groups 2 and 3 biological hazards. Physicians who were uncertain about the scenario for healthcare workers caring for young children (N=6), childcare workers (N=3), and animal handlers (N=9) underscore the inherent complexity and variability involved in evaluating biological risks during pregnancy. Their hesitation may stem from incomplete information about individual immunity, fluctuating epidemiological patterns, or the practical challenges of consistently applying control measures across diverse work settings. In line with the precautionary principle articulated in the cited Spanish guidance (41) and Directive 2000/54/EC (21), such uncertainty underlines the need for case-by-case evaluation, enhanced serological screening, and, where doubt persists, implementation of additional protective measures or temporary reassignment until a definitive risk profile can be established.

Physicians who disagreed with proposed assessment (three in the scenario for healthcare workers, none for childcare workers, and eleven for animal handlers) reflect a profoundly cautious perspective rooted in the recognition of potentially severe foetal outcomes and practical limitations of risk mitigation. Their reluctance likely arises from concerns that standard infection control measures may not fully prevent maternal exposure to pathogens with high teratogenic potential, particularly in settings where subclinical transmission is common or where zoonotic reservoirs render environmental contamination difficult to control. This stance is consistent with the precautionary principle given in the Spanish guidance (41) and Directive 2000/54/EC (21), which mandate that, to avoid any risk to the foetus, work should be reassigned or suspended whenever effective elimination of exposure cannot be guaranteed.

Physicians who agreed with the proposed assessment (N=25, N=27, and N=9 respectively) show confidence in the effectiveness of current preventive frameworks for managing groups 2 and 3 reproductive biological hazards. They appear to trust that a combination of early serological screening, documented immunity (through vaccination or prior infection), and rigorous application of standard infection control measures (including hand hygiene, personal protective equipment, and environmental cleaning) suffices to reduce the risk to an acceptable level. This perspective aligns with the risk management principles outlined in the Spanish guidance (41), which endorses tailored adaptations of work practices when exposure cannot be entirely eliminated, and with the Directive 2000/54/EC (21), which permits occupational exposure to groups 2 and 3 agents under controlled conditions.

High seropositivity rates for CMV, parvovirus B19, rubella virus, VZV, and *T. gondii* among women of reproductive age are largely owed to vaccination or natural infection in childhood or adolescence. The resulting immunity is essential for mitigating risks of severe foetal disease during pregnancy. Occupational titles and general job descriptions alone are insufficient to determine the true level of exposure or immune status for pregnant workers. It is therefore essential to run a comprehensive and individualised risk assessment that will include serological testing, detailed evaluation of specific workplace exposures, and consideration of personal health factors. By leveraging this personalised approach, seropositive pregnant workers can safely continue their work under standard preventive measures, while seronegative individuals or those lacking definitive immunity or exposure data can be reassigned to lower-risk tasks or granted temporary protective leave. This strategy maximises both workplace continuity and maternal-foetal safety in full accordance with the principles articulated in the Directive 2000/54/EC.

### Physical exertion

#### Legal framework

According to the Croatian ordinance on jobs with special working conditions (5), jobs that require heavy physical exertion are contraindicated during pregnancy. Jobs with heavy physical exertion for women are defined as those that require manual lifting or carrying of loads heavier than 15 kg and jobs that are performed predominantly in non-physiological and forced body positions.

Another ordinance, the one concerning protection of workers exposed to static dynamic, psychophysiological, and other strain at work (42) stratifies the risk of physical exertion into low, slightly increased, substantially increased, and high risk (Table 3). Although this bylaw takes into account workload adjustments for female workers, it does not explicitly assess the risk for pregnant workers. At the same time, only the ”substantially increased” and high risk scores range are characterised as heavy physical workload, and as such contraindicated for pregnant workers according to the Ordinance on jobs with special working conditions (5). This means that a wide range of risk scores within the legal framework could be deemed acceptable for a pregnant worker and fail to recognise adverse effects on the health of pregnant worker and unborn child.

**Table 3:** Risk level according to the Ordinance on the protection at work of workers exposed to static dynamic, psychophysiological and other strain at work (42) for lifting, holding, carrying, or pulling and pushing loads

**Risk level**	**Risk score (RS) range**	**Risk of physical exertion**	**Explanation regarding physical overload**
**1**	<10	**low**	Physical overload is unlikely. No health risk is to be expected.
**2**	10–24*	**slightly increased**	Physical overload is possible for less resilient workers.* Workplace adjustment would be helpful for them.
**3**	25–50**	**substantially increased**	Physical overload is also possible for normally resilient workers. Workplace adjustment is advisable.
**4**	>50**	**high**	Physical overload is likely. Workplace adjustment is necessary

* Less resilient workers include people over 40 or under 21 years of age, workers who have just started working (inexperienced workers), or people suffering from a disease.

** Risk score >40 is considered heavy physical exertion and are contraindicated in pregnant workers (5)

However, medical literature indicates an increased risk of adverse pregnancy outcomes in pregnant women whose work requires heavy lifting, prolonged standing, or heavy physical workload (43,44,45). Recommendations are that pregnant workers should not lift a single load heavier than 10 kg or more than 100 kg a day (43,44,45), that is, the frequency of heavy lifting should not exceed 10 times a day (43). Secondly, pregnant workers should not be standing for more than 3 h (45) or 4 h a day (44). The same goes for walking (no more than 4 h a day), bending (no more than 1 h a day), lifting loads above head, and lifting loads from the floor level (43,44,45,46).

A recent meta-analysis (44) has shown that lifting loads of 11 kg or more and lifting more than 100 kg a day is associated with increased risk of adverse pregnancy outcomes, while this association was not observed for loads of up to 5 kg (43, 47). Therefore, lifting single loads of up to 5 kg for majority load lifting patterns throughout pregnancy is recommended as safe in pregnancy (46), and lifting 10 kg or above is not (43, 45). However, there is not enough information to draw conclusions for loads between 5 kg and 10 kg, and this range should be subjected to individual risk assessment, taking into account the limitations of the studies included in meta-analyses and the fact that physical exertion includes other parameters besides load lifting, such as body posture, carrying distance, characteristics of the load, and anthropometric characteristics of the worker (41, 42, 48). Given all loads of up to 10 kg present the lowest risk of physical exertion (level 1), we suggest that risk levels 2 and above are excessive for pregnant workers, although this is not explicitly stated in the respective Croatian ordinance (5).

Even the risk assessment for manual lifting, holding, and carrying of loads proposed by the German Key Indicator Method (KIM) method (49) implies that the boundaries between the risk ranges are fluid because of individual working techniques and performance conditions and that risk classification should be regarded as orientational, with the assumption that the probability of physical overload will increase as the risk scores rise. There are some authors (41, 46) who take into account other parameters such as gestational weeks and load manipulation zones, but the current Croatian legal framework (50) does not regulate time or the procedure for individual risk assessment.

To assess the risk of repetitive manual work tasks with loads of up to 5 kg (42) it is necessary to take into account body posture, especially prolonged standing, since standing for more than 4 h increases the risk of some adverse pregnancy outcomes (44, 45). Considerations should also include the risk of overuse syndromes of the upper limbs such as carpal and Guyon's tunnel syndrome (51, 52).

#### Proposed risk assessment criteria

To calculate the acceptable total workload for pregnant workers using lifting, holding, and carrying loads as examples, we relied on the Key Indicator Method (KIM) adapted from the German Federal Institute for Occupational Safety and Health (48) and embedded in the Croatian Ordinance on the protection at work of workers exposed to static dynamic, psychophysiological, and other strain at work (42).

The risk score (RS) for total workload was calculated from the equation:

RS=(T2+T3+T4)×T1

where T1 is the combined score for total daily lifting frequency, total daily duration of the work task, and/or total daily carrying distance, T2 the score for effective load mass, T3 the score for body position and load during activity, and T4 the score for environmental working conditions and load characteristics.

If we consider medical literature, recommendations for adjusting load handling for pregnant workers, T2 can be either 1 (<5 kg) or 2 (5–9 kg), T3 also 1 (straight upper part of the body without rotation, load positioned close to the body during activity) or 2 (upper part of the body is slightly bend forward or slightly rotated, load during activity is close to or slightly away from the body). Higher T3 scores are excluded, since they involve deep bending or bending forward with the rotation at the same time and loads far away from the body or above the shoulder level, which are not recommended due to anatomical and physiological changes during pregnancy that increase the risk of slips and falls, especially in the later stages of gestation (46, 53). The T4 score should be 0 (enough space for movement, no physical obstacles, solid floors in the same levels, good conditions for load grip), since higher scores involve unfavourable ergonomic conditions, confined spaces, and conditions in which body stability is impaired due to uneven floors, which increases the risk of injury (5, 46, 54). Considering that the sum of acceptable T2, T3, and T4 scores should not be higher than 4, and that T1 can be either 1 or 2, if the product of the single load mass and the total daily repetition does not exceed 100 kg, the total risk score should be <10, signifying level 1 risk, where physical overload is unlikely, and no health risks are expected. A similar assessment could be made for pulling and pushing. In our proposed risk assessment we took this level 1 risk as acceptable for pregnant worker's health and health of an unborn child in case of lifting, holding, or carrying loads or pulling and pushing.

The proposal was accepted by 30 workshop participants, rejected by four, and three were unsure. Those who were against said that even a single 10 kg load is excessive for pregnant worker and that the risk is acceptable only if the single load mass is no more than 5 kg. Some called for individual risk assessment for each pregnant worker.

The proposed algorithm for collecting data needed to reach a conclusion was that the employer should provide the assessing OHP the statement from the workplace risk assessment document which includes the procedure of calculating exposure to static dynamic loads according to the bylaw (42). If the statement is uninformative or incomplete, the OHP should request additional information to calculate the risk level using the original KIM form (48) or the adapted version from the Ordinance on the protection at work of workers exposed to static dynamic, psychophysiological and other strain at work (42). This information should include:

- load mass;
- daily lifting frequency;
- daily total duration of work task;
- total daily distance;
- position of body and load during activity;
- environmental working conditions and load characteristics;
- positioning precision and load speed.

To conclude, the risk level 1 for total workload is the only acceptable risk for pregnant workers whose tasks involve manual lifting, holding, or carrying loads, or pulling and pushing, while the risk levels 2–4 represent physical overexertion. The mass of a single load should be as low as possible and never exceed 10 kg; daily lifting frequency should not exceed 10 times; total daily load should not exceed 100 kg; and standing should not exceed 4 h/day. Individual risk assessment is recommended wherever possible. The assessing OHP should consider making a workplace tour as necessary.

## CONCLUSION

The protection of pregnant workers at their workplace is an issue that has raised political, economic, demographic, and sociological debates across the EU and is much broader than the limited medical framework we propose here. Risk assessment criteria for pregnant workers are not clearly established and harmonised between the EU member states, and guidelines for implementation of relevant occupational health and safety practices are insufficient or missing. It is clear that different workplace hazards require different approaches to risk assessment, which further complicates setting up generally acceptable risk assessment criteria. Beside medical points, including opinions from the operating national medical experts, our proposal is in line with the current national and EU legal framework for pregnant worker protection. We hope that this proposal will contribute to further discussion and harmonisation of expert opinions on the national and EU level and speed up the implementation of Croatian guidelines on workplace risk assessment for pregnant workers. Given the shared challenges, future research efforts should include a pan-European comparative analysis in the member states regarding the medical and legal framework in risk assessment of pregnant workers in order to establish more uniform, detailed, legally and scientifically based protocols for the protection of pregnant workers.
